# Majority rules: how good are we at aggregating convergent opinions?

**DOI:** 10.1017/ehs.2019.6

**Published:** 2019-05-31

**Authors:** Hugo Mercier, Olivier Morin

**Affiliations:** 1 Institut Jean Nicod, PSL University, CNRS, Paris France; 2 Max Planck institute for the Science of Human History, Jena, Germany

**Keywords:** Majority rules, informational conformity, cultural evolution, herding, informational dependency

## Abstract

Mathematical models and simulations demonstrate the power of majority rules, i.e. following an opinion shared by a majority of group members. Majority opinion should be followed more when (a) the relative and absolute size of the majority grow, the members of the majority are (b) competent, and (c) benevolent, (d) the majority opinion conflicts less with our prior beliefs and (e) the members of the majority formed their opinions independently. We review the experimental literature bearing on these points. The few experiments bearing on (b) and (c) suggest that both factors are adequately taken into account. Many experiments show that (d) is also followed, with participants usually putting too much weight on their own opinion relative to that of the majority. Regarding factors (a) and (e), in contrast, the evidence is mixed: participants sometimes take into account optimally the absolute and relative size of the majority, as well as the presence of informational dependencies. In other circumstances, these factors are ignored. We suggest that an evolutionary framework can help make sense of these conflicting results by distinguishing between evolutionarily valid cues – that are readily taken into account – and non-evolutionarily valid cues – that are ignored by default.

## Graphical abstract

**Figure:**
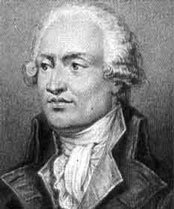

**Social Media Summary:** People are good at taking majority opinions into account, when the information is presented naturally.

Humans rely enormously on others to obtain information. In many cases, we rely not on a single individual, but on several informants – we seek advice from our colleagues, read different newspapers, ask for a second medical opinion. Sometimes these informants provide divergent advice, sometimes they concur. When several people agree, how much weight should we put on their opinion? We have known at least since Condorcet (1785) that following majority rules – adopting the opinion of the majority of the relevant group – can be a powerful heuristic. However, this heuristic should be used with caution, as it only applies under specific circumstances. The present article reviews the literature on how, and how well, humans use majority rules. Our review does not bear on how groups make collective decisions, but on how individuals take into account majority opinions. Although these two domains are linked, they are distinct: groups can make collective decisions without using majority rules, and individuals can use majority rules without any group decision having to be made, or without even belonging to any group. How individuals take into account the decisions made by others will often influence the group's collective capacity to arrive at the right decision. The rules an individual uses to aggregate the decisions of other individuals may thus be seen as an element of collective decision-making, itself an aspect of group cognition (Hutchins 1996). Here, however, the important phenomenon of collective reasoning only interests us insofar as individuals avail themselves of the information generated by a collective.

First, we briefly look at mathematical and modelling work to establish the factors people should take into account when deciding how much weight to put on the majority opinion. Second, we review the literature to see if humans are able to take these factors into account: social psychology, judgment and decision-making, cultural evolution research, developmental psychology, experimental economics, science communication, and political science. Two fields will not be discussed here: research on non-human animals (for reviews, see, e.g. Claidière and Whiten 2012; Haun *et al.* 2013; Whiten 2019), and non-experimental research (for reasons explained below). Finally, we offer a theoretical framework that attempts to make sense of the divergent findings emerging from this review.

Because this article draws on several different studies, it might be confusing to adopt the terminology used in each to refer to the same concepts. Instead, we will rely on the following terms: the *recipient* is the individual who receives the information (typically the participant in experimental studies), the *informants* are those individuals who provide information to the participants. The information provided by informants is referred to as the informants’ *opinions*, and the grounding of these opinions – the pieces of information on which they are based – is referred to as their *source(s)*.

As a final terminological note, the influence of majority rules has chiefly been studied by social psychologists under the label of conformity, distinguishing between normative conformity – aiming at ingratiation with the group one conforms to – and informational conformity – aiming at accuracy (Deutsch and Gerard 1955; for an attempt to experimentally disentangle the effects of both types of conformity, see Sowden *et al*. 2018). It is often difficult to tell exactly how much these two types of conformity are engaged in a given task – a difficulty linked to the fact that the theoretical distinction is itself not entirely clear-cut (see Cialdini and Goldstein 2004; Coultas and van Leeuwen 2015; Deutsch and Gerard 1955; Hodges 2015). The difficulty in distinguishing informational and normative conformity is even more acute in the case of non-experimental studies, which is the main reason why we have chosen to exclude them from the present review.

In spite of these difficulties, we should note that normative and informational conformity serve different functions: to be accepted by a group for the former, to form accurate beliefs for the latter. We can thus reasonably expect that distinct cognitive mechanisms would have evolved to fulfil each function, taking into account different cues, performing different computations, and yielding different outcomes. As a result, throughout the review, we have attempted to focus on studies that are more likely to elicit informational rather than normative conformity, as we are primarily interested in how people use majority rules to gain information. This means for instance that we have focused on experiments in which the answers are given in private (for evidence that such settings are less likely to elicit normative conformity, see Asch 1956; Bond 2005; Corriveau and Harris 2010; Haun and Tomasello 2011), the answers can be more or less accurate (in contrast with arbitrary conventions), and in some cases at least, there are incentives for accurate answers.

## 1. When should majority rules be followed?

The basic result supporting the rationality of following majority rules is that if each informant is more likely to be right than wrong then, as the number of informants that concur increases, their chances of agreeing on the correct answer increase.

The extension of this basic result that has played the most important role historically is the Condorcet Jury Theorem (Condorcet 1785). The Condorcet Jury Theorem was developed in the context of voting, but what matters is not the formal process of voting, but simply that several informants state their opinions, and that these opinions are dichotomous. The Condorcet Jury Theorem demonstrates that, providing some conditions are met, the chances that the majority supports the correct choice increase with the total number of informants, and converge towards 1 as the number of informants increases. As a result, the first two factors that should be taken into account when deciding whether to use majority rules are the relative and absolute size of the majority (i.e. how consensual is the majority opinion within a given group, and how large is the group in which this opinion is accepted by the majority).

Analytically, the extension of the Condorcet Jury Theorem to cases in which there are more than two opinions has raised some well-known issues. In particular voting paradoxes, in which no opinion is supported above the others, become a possibility. Still, simulations strongly suggest that, when more than two options are available, plurality rules – in which one follows the opinion supported by a plurality of informants – perform very well for a wide range of parameters (Hastie and Kameda 2005).

Majority rules, understood as following the most common opinion in a group, thus have strong rational grounding. Another potential advantage of following majority rules is their simplicity (Boyd and Richerson 1985). Compared with other means of information aggregation, majority rules can take a form that is computationally trivial, only requiring a counting of the opinions without any further weighing of the individual opinions. Moreover, majority rules sometimes outperform more sophisticated mechanisms of information aggregation (Hastie and Kameda 2005), making majority rules one of those ‘simple heuristics that make us smart’ (Gigerenzer *et al*. 1999).

For all their power, majority rules should not be blindly followed. There are well-known factors that limit their applicability. The most obvious constraint on majority rules is that the informants must, on average, be right rather than wrong. This is often framed in terms of competence. However, informants can also provide wrong information intentionally or through negligence (see, e.g. Austen-Smith and Banks 1996). To be able to rely on majority rules, one should thus first ensure that the informants are competent and benevolent.

Another constraint on majority rules is that in many, probably most, cases, recipients of social information have a preconceived view regarding the opinion at hand. These priors should be taken into account, so that the output of majority rules should be less potent when it runs against stronger priors. This simple consequence of the fact that social influence is not the sole source of our beliefs is accepted by all theories of social learning (e.g. Asch 1955; Festinger 1954). Accordingly, cultural evolutionary theorists show that purely social learning as distinct from purely individual learning is less likely to evolve when the latter is costly and uncertain owing to a fast-changing environment (Boyd and Richerson 1988). For individuals, ‘copy when uncertain’ is a sensible heuristic (Laland 2004).

These factors – that the informants should be competent and benevolent, that the opinion should be weighted against the recipient's priors – are not specific to majority rules. The only factor that is specific to majority rules is that the opinions of the members of the majority should have been acquired independently – i.e. that there should be no informational dependencies between the opinions. Consider the case of two friends, Lana and Patricia, whom you both trust, telling you that Justin, an acquaintance of yours, is rude. How much weight you should give to your friends’ opinions depends on how independent they are. If the only reason Patricia believes Justin to be rude is because Lana told her so, then you should largely discount Patricia's opinion (not entirely, because Patricia's endorsement of Lana's opinion suggests that she has independent reasons to trust Lana in such matters; see Estlund 1994). Similarly, if both Patricia and Lana ground their opinions on witnessing the exact same behaviour (say, one time when Justin was very rude to a waiter), then their combined opinion does not weigh much more than their individual opinions. In contrast, if Patricia and Lana ground their opinion of Justin on non-overlapping observations, then the convergence in their opinions provides more grounds for accepting that Justin is rude (for a formal analysis of the role of causal dependencies in the Condorcet Jury Theorem, see Dietrich and Spiekermann 2013).

More recent modelling work has confirmed the robustness of Condorcet's original insight, for instance by looking at the trade-offs between personal and social decisions (King and Cowlishaw 2007). Other work has extended the original model, for example by modelling the influence of the threshold (in terms of relative majority) on whether agents follow majority decisions (MacCoun 2012). Finally, a few other studies appear to contradict the Condorcet Jury Theorem. For example, Kao and Couzin (2014) have shown that, when the environment is particularly complex, small groups might be more likely to make correct decisions than larger groups – so that the absolute size of the majority would not be a sound cue anymore. However, it is likely this apparent reversal is caused by issues of informational dependency, which would make it consistent with the classical Condorcetian framework. Thus, even though we have not presented an exhaustive review of mathematical and modelling work on majority rules, the literature appears to support the Condorcetian framework, with some potential minor deviations.

To summarize, in order to aptly use majority rules, recipients should pay attention to the following factors: (a) relative size of the majority; (b) absolute size of the majority; (c) competence of the informants; (d) benevolence of the informants; and (e) degree of informational dependency between the opinion of the informants.

Before reviewing the experimental literature looking at whether people take these cues into account, we must specify that majority rules are only one of the factors deciding how much weight people give to others’ opinions: people also look at the content of the opinion and of the arguments supporting it (for reviews, see Mercier 2017; Sperber *et al*. 2010). In some cases, these other factors trump majority rules, as when the correct answer to a logical problem is defended by only one individual against many individuals agreeing on the incorrect answer. In such a situation, if the individual defending the correct answer is able to produce good arguments in its support, she will nearly always prevail against the majority (Laughlin 2011; Trouche *et al*. 2014; for slightly different problems, see Laughlin *et al*. 2006). However, here we focus on factors that affect how people use majority rules, not how majority rules are weighted against other, independent factors.

## 2. Review of the experimental literature

### 2.1. Absolute and relative size of the majority

Starting with the effect of absolute group size, several studies using different paradigms and participants of different ages have found that the absolute number of informants supporting an opinion can influence how much participants take this opinion into account. Even though some studies compare the influence of one vs more than one informant, we will use ‘group size’ to refer to the number of informants, even when there is only one informant. The most robust result is probably that obtained through a meta-analysis of the experiments that have collected private answers in the Crutchfield paradigm (Bond 2005). In this paradigm, participants usually complete a non-trivial perceptual task and their answers are private, in contrast with the standard Asch paradigm (Crutchfield 1955). Participants are provided with a signal that the experimenter presents as the answers of one or more other participants (informants). In this context, participants are more likely to adopt an answer defended by more informants (Bond 2005). This effect of absolute group size is found not only through the meta-analytical comparison of several studies with different group sizes, but within individual studies that have manipulated group size (Gerard *et al*. 1968; McElreath *et al*. 2005; Morgan *et al*. 2012; Toyokawa *et al*. 2018; Wolf *et al*. 2013). Tanford and Penrod's (1984) meta-analysis, although it includes mostly Asch-like studies where participant choices are public, does find the expected effect of absolute majority sizes, even after controlling for the publicity of responses.

An effect of absolute group size has also been observed in young children. When presented with perceptually ambiguous items, 3- and 4-year-olds are more likely to change their mind regarding the identity of the items when confronted by three unanimous informants than by a single informant (Bernard *et al*. 2015).

Other experiments, however, have failed to observe an effect of absolute group size on informational conformity. These failures probably stem from different factors. One factor might be that the experiment mostly engaged normative conformity. For instance, one experiment observed whether participants would engage in an unusual behaviour as they witnessed others engaging in this behaviour (Coultas 2004). Such an experiment is likely to tap into normative conformity: the behaviour is public, and there is no question of accuracy. This experiment found effects of relative group size, but not of absolute group size. These results are consistent with normative conformity, for which what mostly matters is whether the opinion is unanimous or not, rather than the absolute group size (see, e.g. Asch 1955). Other factors explaining the lack of effect of absolute group size are floor and ceiling effects. As an example of ceiling effect, in an experiment the perceptual task was made very difficult by lowering the amount of attention people could give the stimuli (Campbell and Fairey 1989). When participants were confronted with the opinion of a single informant, a large majority of the participants adopted it. It is not surprising then that an also high number of participants adopted an opinion defended by three informants. Conversely, Efferson *et al*. (2007; see also Efferson *et al*. 2008, 2016) found that participants, in a task aimed at simulating foraging decisions, did not take any heed of the answers given either by several informants or by a single informant – an example of a floor effect (possibly because their priors were too strong).

Turning to the effects of relative group size, a series of experiments, which involved different tasks with clear accuracy goals, systematically varied the proportion of informants agreeing on a given answer, from 50 to 100% (Morgan *et al*. 2012). The odds that the participants would adopt the answer supported by the majority increased with the relative size of the majority. Similar results have been observed in other experiments using a variety of materials – although some of these experiments might also have tapped into normative conformity (Claidière *et al*. 2012; Coultas 2004; Eriksson and Coultas 2009; Weizsäcker 2010). Using a perceptual task (dot-discrimination), the same result was clearly observed among 6- and 7-year-olds, but not among younger children (Morgan *et al*. 2015, p. 201).

The exact shape of the relationship between the size of a majority and the propensity to follow it is a matter of debate. On the one hand, social psychologists from the Social Impact Theory school (e.g. Eriksson and Coultas 2009; Latané 1981; Latané and Wolf 1981) have argued that the minority has a stronger impact, in proportion to its relative size, than the majority. This (in Eriksson and Coultas’ formulation) implies that the function relating the probability of following a given choice (assuming a binary choice between two options) to its frequency in a population of informants assumes the shape of an inverted ‘s’. In other words, majorities tend not to be followed in proportion to their size, but a little less. The opposite view has also been defended, under the name ‘conformist transmission’, also sometimes called ‘majority bias’ (Boyd and Richerson 1996; Henrich and Boyd 1998). In that view, individuals follow the majority choice in a biased and disproportionate way, that is to say, out of proportion with the majority's (relative) size. This time, the relation between a majority's relative size and the probability that it will be followed forms an s-shaped curve. There is currently no decisive empirical support in favour of either of these two conflicting views, as distinct from the more straightforward hypothesis of a linear or logarithmic relation (neither s-shaped nor inverted-s-shaped). Experimental results on human adults have yielded discrepant and inconclusive findings (Coultas 2004; Efferson *et al*. 2008; Eriksson and Coultas 2009; McElreath *et al*. 2008; Morgan *et al*. 2012; Muthukrishna *et al*. 2016). Studies of jury decisions in US trials (although not comparable with controlled experiments on many grounds) appear to support a model of influence where relative majority size impacts social influence in a non-linear fashion, minorities having an influence greater than their numerical weight, with no threshold effect at 50%, in violation of conformist transmission (Tanford and Penrod 1984).

In the advice-taking literature, participants are typically asked to take into account numerical estimates – for instance how many calories there are in an apple – which means that few informants would agree on any single estimate. As a result, researchers rely on a different measure of convergence: the degree of agreement among informants, rather than how many informants agree on the exact same estimate. It has been repeatedly observed that high agreement among several informants makes participants more likely to move their own estimates towards that of the informants (Budescu *et al*. 2003; Budescu and Yu 2007; Harries *et al*. 2004; Yaniv 1997; Mannes 2009; see also, in the context of herding in finance, Barron and Stuerke 1998).

Instead of directly providing information from several informants, other experiments have provided numerical data regarding the majority size – say, 75% of citizens are in favour of this policy and 25% disapprove. Most notably, research conducted in political science has studied the effects of presenting polling data on political attitudes. These experiments do not reveal a consistent effect of majority information. Participants sometimes veer towards the majority (bandwagon effect), sometimes towards the minority (underdog effect) (for review, see Mutz 1998; for similar research in social psychology, see, e.g. Petty and Cacioppo 1986). Many experiments have provided majority information along with arguments supporting either the majority opinion or the minority opinion, or both. These experiments have also yielded conflicting results, revealing several moderating variables such as argument quality and the participants’ prior beliefs (for review, see Martin and Hewstone 2008). In some cases, argument quality completely trumps majority information, so that a strong argument provided by a minority is very likely to be accepted, whereas a weak argument provided by a majority is nearly never accepted (Claidière, Trouche, and Mercier 2017; Laughlin 2011; Trouche *et al*. 2014).

A recent study has looked at whether participants have some understanding of the advantages of voting demonstrated by the Condorcet Jury Theorem (Mercier, Dockendorff, and Schwartzberg, submitted). Participants are asked to estimate the chances that, if a majority of members of an assembly voted for one option (out of two), the option selected was the best. Several relevant factors (size of the majority, competence of the members, etc.) are manipulated, and the results show that, although the participants are able to take some relevant factors into account, they consistently underestimate the odds that the majority selects the best option.

Finally, several studies in science communication have tested whether providing participants with information about the degree of scientific consensus affects their opinions on topics that have proved controversial among the general public (climate change, vaccination and GMOs). In these studies, consensus information is presented in different ways: the percentage of scientists who agree is provided (Deryugina and Shurchkov 2016; Dixon 2016; Dixon *et al*. 2017; Kerr and Wilson 2018; Landrum *et al*. 2018; Lewandowsky *et al*. 2013), but it can be accompanied by other modes of presentation, such as pie charts, pictures of groups of scientists or metaphors (van der Linden *et al*. 2014, 2015a, b). Presenting consensus information is sometimes effective in moving participants’ opinions towards the consensus (Deryugina and Shurchkov 2016; Dixon 2016; Kerr and Wilson 2018; Lewandowsky *et al*. 2013; van der Linden *et al*. 2014, 2015a, b), but not always (Dixon *et al*. 2017; Landrum *et al*. 2018).

### 2.2. Competence and benevolence of the informants, prior beliefs of the recipient

Here we focus on factors that are not specific to informational conformity, since they consist of individual characteristics, whether they are informant characteristics (competence and benevolence) or recipient characteristics (prior beliefs). These factors have been shown to play an important role in how people evaluate opinions coming from a single informant (Bonaccio and Dalal 2006; Petty and Wegener 1998; Yaniv 2004; for review, see Mercier 2017). In contrast, few experiments have directly tested the effects of informant characteristics on informational conformity. Andersson *et al*. (2014) found that asking participants to focus on the accuracy of several informants who happened to have been inaccurate led participants to appropriately discount the opinion of these informants. Bernard, Proust, and Clément (2015) found that 6-year-olds – but not 4- or 5-year-olds – adequately discounted convergent opinions when the informants had been inaccurate in the past. Efferson *et al.* (2016) found that participants were more likely to copy convergent informants that were faced with the same task and the same payoffs as themselves. Still, in spite of these limited data, we should expect that informant characteristics which are known to be taken into account when processing information from single informants should keep playing their role when processing information from multiple informants.

Several experiments have shown that the prior beliefs of a recipient modulate the way she uses majority rules. It has been consistently found that, the less participants are sure of their own opinions, the more likely they are to adopt the majority opinion. This is true of comparisons across different paradigms: for instance, when answers are private, the group opinion is nearly entirely neglected in the Asch paradigm, while the group opinion has a strong influence in more difficult perceptual tasks (Bond 2005). This result has also been obtained by experimental manipulations of task difficulty, for a variety of tasks (Baron *et al*. 1996; Campbell and Fairey 1989; Morgan *et al*. 2012; Toyokawa *et al*. 2018). Similarly, participants who form their opinions on the basis of more variable information, and thus form less reliable opinions, are more likely to adopt the opinion defended by a majority of informants (Andersson *et al*. 2014; McElreath *et al*. 2005; see also Antenore *et al*. 2018). Children as young as 3 years of age have been found to be influenced by convergent informants to a greater extent when they face a difficult perceptual task, relative to an easier version of the task (Bernard *et al*. 2015; see also Morgan *et al*. 2015).

The influence of recipients’ prior beliefs is, on the whole, positive: it makes for a finer-grained discrimination and allows for improved performance (see, e.g. Morgan *et al*. 2012, 2015). However, participants often put too much weight on their prior beliefs, displaying egocentric discounting. Studies of social influence often find that participants holding the right answer to a question rationally exhibit higher confidence and lower susceptibility to social influence (see, e.g. Jayles *et al*. 2017). Egocentric discounting is different: it is the tendency of recipients to put more weight on their own opinion, relative to the opinion of another informant, even when they have no reason to believe they are more competent than the informant (Trouche *et al*. 2018; Yaniv and Kleinberger 2000). Egocentric discounting is apparent in various economic games involving convergent social information, where participants spurn the information manifested in other people's choices, even though it would be beneficial for them to take it into account (Efferson *et al*. 2007, 2008; Mannes 2009).

The clearest example is perhaps provided by the experimental literature on information cascades (Anderson and Holt 1997). In information cascade experiments, participants have to decide which colour is more common in the balls drawn from an urn. Participants base their opinion on a single draw that only they have access to, as well as on the actions exhibited by the sequence of all previous participants. The standard models for this experiment assumes agents to be competent and not intent on misleading others, an assumption that holds in laboratory settings (Bikhchandani *et al*. 1998; Goeree *et al*. 2007).

In the Bayes–Nash model of information cascades, the way that participants should weigh the information given by other participants’ behaviour is straightforward. If the choices of the last two players who made a move before me do not coincide, what is optimal is to follow my private information. If the choices of the last two participants coincide, on the other hand, the optimal choice is to copy them, regardless of my private information (the colour of the ball I see). In the latter case, it is possible for the wrong opinion to take hold in a group, if the first few participants’ draws are misleading (i.e. they drew the ball from the rare colour). Other participants should then bet on the rare colour, even if they themselves have drawn a ball from the common colour, potentially creating a negative information cascade. However, the opposite (a positive information cascade where players choose the common colour) is much more likely. Whether the cascade is positive or negative, however, a rational participant, not knowing whether past participants were right or wrong, should follow it (in this model).

One important finding from the experimental literature is that both negative and positive cascades are much less frequent than this Bayes–Nash model implies (Goeree *et al*. 2007). This deviation from the normative model occurs for two reasons. First, there is a substantial element of randomness in the participants’ choices. Second, participants overweigh their private information, instead of following the informants, in the situation where the private signal they receive contradicts the choices of the previous participants (Goeree *et al*. 2007; Weizsäcker 2010; Ziegelmeyer *et al*. 2013). Egocentric discounting is not detrimental to participants when it breaks a negative cascade, but it is when it makes them depart from a positive one (which is to say, most of the time).

Information cascades, in conjunction with the other experimental studies reviewed above, demonstrate that recipients are able to weigh informational conformity as a function of various informant characteristics and of their prior beliefs. This weighing likely improves performance. The main bias in this weighing is a tendency for recipients to put too much weight on their prior beliefs, relative to the convergent opinion of a small number of informants.

### 2.3. Informational dependencies

Informational dependencies occur when the opinions of several informants are confounded. This can occur because the informants’ opinions have partially or entirely identical sources (common sources), because some informants had contact with one of the other informants’ opinions before forming their own (contact), or because they shared an extrinsic motivation to express the same opinion (external pressure). An extreme case of dependency would occur when several different opinions find their origins in one single individual (see Morgan *et al*. 2012). Informational dependencies can be explored experimentally in two ways. The first is by providing participants with general cues of dependency: correlations between the informants that may or may not indicate common sources, contact or external pressure, without giving any direct evidence of either. The second is by providing specific cues of correlation, contact or external pressure. The results differ depending on the cues’ specificity.

#### General cues of dependency

A number of studies have examined how participants weigh the opinions offered by several informants as a function of how correlated these informants’ opinions are or have been in the past. Information about correlation has been presented in different ways: (a) as an explicit correlation (e.g. some ‘subjects were told that the correlation among forecast errors was approximately +0.8 for all forecaster pairs’; Maines 1990, p. 36; see also, Kroll *et al*. 1988); (b) implicitly – the opinions happened to be based on more or less overlapping information (a fact that was unknown to the participants and could only be inferred through the distribution of the opinions) (Budescu and Yu 2007); (c) as a list of previous opinions from the same informants, opinions which were manipulated to be either highly correlated or largely uncorrelated (Andersson *et al*. 2014; Maines 1996; Votruba and Kwan 2015); and (d) as information about how the informants were selected, in particular telling participants that some informants were selected because they were likely to agree with one another (Yaniv *et al*. 2009). These methodologies yield convergent results: participants robustly fail to take correlations between the informants’ opinions into account (for similar failures regarding non-social sources of information, see Slovic 1966; Soll 1999).

#### Specific cues of common source

Whalen *et al*. (2018) show that participants are more likely to trust the opinion of three informants (over their own private information) when the three informants have access to one independent piece of evidence each, rather than to one piece of evidence common to all three. Note, though, that participants’ endorsement of the informants’ opinion, in the latter case, is higher than it should normatively be, consistent with the ‘multiple informants’ effect (Harkins and Petty 1981). Relatedly, participants discount information from a single informant when this informant bases her opinion on the same information as the participants, according to Gonzalez (1994).

Mercier and Miton (2019) found similar results whether the common source was hearsay (i.e. the opinions were based on hearsay from the same source) or perception (i.e. the opinions were based on having perceived the same event). One study, in contrast, exposed participants to direct cues of common sources, with no effect. The participants were provided with several newspapers articles, issued by distinct newspapers, that either all relied on the same expert or on different experts (Yousif *et al*. 2018). Participants did not put more weight on the opinion defended by different experts (possibly because they interpreted the endorsement of different newspapers as a sign of greater expertise; see Estlund 1994).

#### Specific cues of external pressure

In a study by Conway and Schaller (2005), participants were made to play the part of an executive in a firm who had the final say in an important purchase decision. In the control condition, each participant witnessed all their colleagues choosing option A over option B independently, whereas in the experimental condition, the colleagues were all ordered to choose option A by their common boss. Participants were more likely to choose option B in the latter case, suggesting that they discounted the opinions that reflected external pressure. Similarly, Mercier and Miton (2019) found that participants put less weight on the convergent opinions of informants when the informants shared the same motivation in expressing the opinion (i.e. to say something positive about a restaurant owned by a friend).

#### Specific cues of contact

Most experimental manipulations of informational dependencies focus on contact: the informants’ exposure to the other informants’ opinions. These experiments can be sorted according to the strength of the evidence, which can be *weak* if it leaves open the possibility that the informants formed their opinions on the basis of independent evidence, or *strong* if most informants clearly derived their opinions from hearsay from another informant only.

#### Weak cues of contact

In one experiment, participants were provided with the opinions of several informants. In one condition, participants were told that each of the informants had seen the other informants’ respective opinions before forming their own final opinion. In the other condition, participants were told that the informants had had no access to the other informants’ opinions before forming their own. Participants put more weight on the opinions of the informants in the latter condition (Bloomfield and Hales 2009). In another experiment, participants were provided with the opinions of two informants, and they were told either that the opinions had been formed independently or that one informant had seen the other's opinion before forming hers. Participants were less influenced by the second informant's opinion when this informant had seen the opinion of the first informant (Keshk 2012). However, this discounting was only observed when participants were motivated to disregard the informants’ opinion. Relatedly, Wilder (1977) showed that four multiple informants were less persuasive when they were described as one group of four, rather than two groups of two, and even more persuasive when they were described as four individuals.

Harkins and Petty (1987) presented students with arguments about the organization of an academic exam. In one condition, the informants providing the arguments were presented as three students reflecting independently. In another condition, the arguments were described as the work of a committee formed by three students. Participants were less persuaded by the committee's arguments than by the three individual students. More precisely, the committee persuaded them no more than a single student did. Lopes *et al*. (2007) likewise show that a group's opinion is considered more valid when its members are described as having heterogeneous information. Hess and Hagen (2006) found that a piece of gossip was judged more believable when it came from an independent informant, as opposed to someone who merely reported and endorsed the gossip, or to an independent witness with friendship ties with a concerned party. Recently, Einav (2017) asked children (5- to 9-year-olds) and adults to decide between information offered by two consensual groups. The members of one of the two groups had had access to one another's answers, so that their answers probably suffered from informational dependencies. There was a developmental shift in how the opinions of these two groups were weighted, with younger children favouring the group whose members had seen the other members’ answers, while older children and adults favoured the group whose members had not had such access. This result thus replicates the findings above, and shows their developmental limits.

#### Strong cues of contact

One experiment (with young children) makes it clear to the participants that most informants in a group formed their opinion by contact with other opinions alone. Participants were provided with the contradictory opinions of two groups of informants. Informants from the first group formed their opinions independently – they all had perceptual access to the relevant information. In the second group, one informant had perceptual access to the relevant information, and communicated her opinion to the second informant, who communicated it to the third, etc. When the two groups were of the same size, both adults and 4-year-olds were more likely to endorse the opinion of the first group (Hu *et al*. 2015). However, when the first group comprised four members and the second group six, only adults kept being more likely to endorse the opinion of the first group.

#### Evidence from information cascades

The experimental literature on information cascades (e.g. Anderson and Holt 1997) could be taken as showing that participants do not take into account informational dependencies in a felicitous way. To understand why, we should keep in mind a strange property of the Bayes–Nash model of cascade formation (Bikhchandani *et al*. 1992; Goeree *et al*. 2007). As soon as two consecutive players make an identical move, all of the participants following them should copy that move, irrespective of their private information. Whether the cascade has been followed for two steps or 150 should not matter to participants. Once a chain is started, the copiers that make up the chain should take no account of their own private information, and for this reason their actions are strictly uninformative – they merely reflect the information of the first two players who started the cascade. Participants do not behave as expected by this model, however: they are much more likely to follow a sequence of identical moves when that sequence has been going on for a long time (Kübler and Weizsäcker 2005; Weizsäcker 2010).

From the standard model's point of view, this demonstrates a lack of understanding of informational dependencies. This, however, is not a valid argument, since the Bayes–Nash model is inaccurate on this point. Participants show egocentric discounting (they sometimes fail to follow the cascade when it contradicts their private information). Because of this, a cascade that has lasted for a great number of steps does reflect substantial information concerning the state of the world, and should be given greater weight than a shorter one. Given this, the way participants choose to follow others is close to optimal, if we disregard their bias for egocentric discounting. If participants were not properly sensitive to informational dependencies, they would be prone to following the crowd against their own private information, mistakenly thinking the crowd to be better informed than them in cases where it is not. Participants overwhelmingly avoid this bias. According to Weizsäcker's (2010) meta-analysis, participants behave in a near-optimal fashion in such cases, reaping 75% of the possible rewards (while a perfectly optimal player would reap 76%). This performance stands in sharp contrast with the performance exhibited when it is optimal for participants to contradict their own information, in which case egocentric discounting leads to a loss of rewards.

Overall, specific cues indicating common source, contact or external pressure, are taken into account to a much greater extent than general cues that indicate possible dependencies through correlations, without pointing at a specific cause. Specific cues of common source, contact, or external pressure, seem more efficient when they are strong enough to exclude other causes (for instance, when it is obvious that the informants could *only* have formed their opinions through contact with other informants).

## 3. Conclusion: making sense of divergent findings

At first sight, this review might appear to yield confusing results. In some cases, participants appropriately use a given factor to decide how much they should rely on majority rules. In other cases, they completely overlook a factor. Moreover, the same factor – e.g. whether there are informational dependencies or not – is sometimes taken into account, sometimes ignored.

We suggest that the framework of the evolution of communication (Maynard Smith and Harper 2003) can help make sense of these findings. Communication is known to be a risky endeavour, as recipients can be lied to or otherwise manipulated. Communication is particularly risky for humans, given its importance in their ecology. As a result, it has been suggested that humans have been endowed with cognitive mechanisms whose function is to evaluate communicated information (Mercier 2017; Sperber *et al*. 2010). These mechanisms of epistemic vigilance would take into account a variety of cues and influence which messages we accept and which we reject. Some of these cues pertain to the competence and benevolence of informants, others to the content of the message, in particular in relation with our prior beliefs. A wealth of evidence shows that humans, from a very young age (Clément 2010; Harris 2012; Harris and Lane 2014), are able to appropriately take many such cues into account, so that plausible messages coming from competent and benevolent informants are more likely to be accepted (for review, see Mercier 2017).

The studies reviewed above suggest that these epistemic vigilance mechanisms are also applied to majority rules, so that the opinion of the majority is granted more weight when the informants are competent and benevolent, and when the majority opinion conflicts less with the recipient's prior beliefs. Moreover, an evolutionary framework can account for the main deviation from apparently optimal behaviour observed in this area: the tendency to put too much weight on the recipient's priors relative to communicated information (egocentric discounting). If we consider the uncertainty about informant benevolence, and the relative costs associated with trusting too much vs not trusting enough, a degree of egocentric discounting appears sensible enough (see, Sperber *et al*. 2010; Trouche *et al*. 2018).

At least as much as communication, the use of majority rules is probably evolutionarily ancient: many animals rely on such rules, for instance when making foraging decisions (Conradt and List 2009; Conradt and Roper 2003). Given the importance of group decision-making (Boehm *et al*. 1996) and, more generally, of cooperation and communication in humans, we should expect them to be equipped with cognitive mechanisms dedicated to majority rules. These mechanisms would also pay attention to a variety of cues in order to determine how much weight to grant majority opinions.

However, not all cues are created equal. In particular, we can only expect cognitive mechanisms to have evolved in response to cues that were accessible and reliable in the environment that shaped these cognitive mechanisms – evolutionarily valid cues (e.g. Barkow *et al*. 1992; Cosmides and Tooby 1994; Sperber 2001). The concept of evolutionarily valid cues shares some traits with that of ecological validity (see, e.g., Gigerenzer 2007; Gigerenzer *et al*. 1999), but it stresses the importance of past, in addition to current, environments.

To the extent that the cognitive mechanisms we rely on to deal with majority rules are somewhat well adapted, we should expect them to primarily rely on evolutionary valid cues. This does not mean that we should be unable to learn to rely on other cues: after all, humans are typically endowed with learning mechanisms rather than purely innate mechanisms. Still, efficient learning mechanisms rely on strong built-in biases (e.g. Carruthers 2006; Gallistel and Gibbon 2000). Thus, even though humans can surely learn how to use new cues when dealing with majority rules, we should expect some cues to be more readily taken into account.

Even if we can only offer approximations for the environment that shaped the cognitive mechanisms dealing with majority rules, some plausible assumptions help make sense of which cues are more easily taken into account. Regarding the size of the majority, an evolutionarily valid cue would have been witnessing individuals offering different opinions. In contrast, written numbers, and in particular written statistical indicators (e.g. ‘75% of subjects chose X’) are a recent cultural invention, making them a potentially non-evolutionarily valid cue (e.g. Chrisomalis 2010; Dehaene 1999). The distinction between evolutionarily valid and non-evolutionarily valid cue is reminiscent of the distinction between decisions from experience (here, seeing the individual opinions of different informants) and decisions from description (here, being provided with a numerical description of the majority) (Hertwig and Erev 2009).

The distinction between evolutionarily valid cues and non-evolutionarily valid cues fits the results reviewed in Section 2.1. Participants, including young children, appropriately weigh majority opinion as a function of the absolute and relative size of the majority when they can see the opinions of individual informants. In contrast, participants do not systematically follow majority rules when the information is presented in written numerical format (e.g. ‘75% of the people say’). Moreover, participants have no abstract understanding of the benefits of majority rules – in the same way as people have a good grasp of intuitive physics, but not of theoretical physics (see Mercier 2019).

Turning to informational dependencies, some relevant cues are omnipresent in all known human societies, and these cues can be assumed to have been present in the relevant evolutionary timeframe. In particular, recipients would have had some information about the source of the informants’ opinions. Recipients are sometimes able to infer this information – e.g. having seen Patricia talk to Lana before she talks to you. More often, information about the source of their opinions is volunteered by informants. All languages contain tools with which informants can specify the source of their opinions – evidentiality markers (see Aikhenvald 2004, p. 10ff). Some evidentiality markers are explicit (‘Lana told me’), others implicit. Many languages have evidentials – gramaticalized, mandatory evidentiality markers – so that every assertion has to specify the source of the opinion offered (Aikhenvald 2004; Lazard 2001).

Because people would often have information about the actual or potential sources of informants’ opinions, specific cues of common source, contact or external pressure should be evolutionarily valid, and thus appropriately taken into account. As we saw above (Section 2.3), this seems to be the case. In contrast, general cues of dependency – e.g. a correlation coefficient – should not be evolutionarily valid, and thus they should be largely ignored. Again, this seems to be the case.

That some cues are presumed not to be evolutionarily valid does not mean that they cannot be learned. People can learn to use non-evolutionarily valid cues if they are sufficiently motivated. For instance, observational evidence suggests that financial experts are able to take informational dependencies into account, even when they are not presented in a evolutionarily valid manner (Cooper *et al*. 2001; Jegadeesh and Kim 2010; Kim and Pantzalis 2003; for another kind of expertise, more informal, see Zhou and Guo 2016).

The attempted exhaustiveness of the present review was made possible by restrictions on the range of evidence we considered. First, we focused on situations likely to elicit informational, rather than normative conformity. Normative conformity might dictate very different behaviours than informational conformity (as in, for instance, the Asch conformity experiments). While people are influenced by normative concerns in the public expression of their opinion or behaviour, it is often difficult to tell whether they are also following majority rules properly in the formation of their private opinions. This difficulty is particularly acute in the case of non-experimental studies, which is the main reason why we had decided to exclude them. A potential unfortunate side-effect of this choice was the focus on standard experimental participants, when non-experimental studies might cover a broader diversity of people. Finally, we only looked at how individuals use majority rules to form opinions, not how groups use such rules to make decisions. Although, as we have argued at the outset, the two issues are largely distinct, there is still potential for considerable interplay, which should be the topic of future studies.

In spite of these limitations, the present article offers, to the best of our knowledge, the first integration of the various fields that have conducted experimental studies relevant to the use of majority rules by humans. We have attempted to make sense of these results, first, by organizing them as a function of the various factors that normative models say should be taken into account, and second, by using an evolutionary framework to explain why some cues to the validity of majority rules are sometimes properly processed, and sometimes ignored by participants.

Future work should offer more direct tests of these hypotheses, in particular by contrasting evolutionarily valid and non-evolutionarily valid cues. We predict that evolutionarily valid cues will be taken into account in a way that better fits mathematical models of when majority rules should be taken into account (for some preliminary evidence, see Mercier, Majima, Claidière, and Léone, submitted). This does not mean that non-evolutionarily valid cues will always be ignored, as they can be processed by other mechanisms, merely that they will be taken into account less appropriately. Another prediction of the present evolutionary framework is that there should be less cross-cultural and developmental variation in how evolutionarily valid cues are taken into account. Taking evolutionarily valid cues into account should not depend on schooling or explicit learning. In contrast, making sound use of non-evolutionarily valid cues should often require explicit teaching. Evolutionary approaches can drive more research into similarities and differences across cultures (Apicella and Barrett 2016), a move that would greatly benefit research on the use of majority rules (for some examples of cross-cultural research, see Corriveau and Harris 2010; Van Leeuwen *et al*. 2018). Finally, our evolutionary approach could help better distinguish between normative and informational conformity. As mentioned earlier, both types of conformity serve different functions, which are plausibly fulfilled by different cognitive mechanisms, taking different cues into account. An evolutionary perspective should make predictions regarding which cues to normative conformity are evolutionarily valid. These predictions could then be evaluated against the existing literature and in novel experiments comparing for example the weight granted evolutionarily valid cues with informational vs. normative conformity in different contexts.
